# Effect of Dehydrogenation and Heat Treatments on the Microstructure and Tribological Behavior of Electroless Ni-P Nanocomposite Coatings

**DOI:** 10.3390/ma17225657

**Published:** 2024-11-20

**Authors:** Giulia Pedrizzetti, Enrico Baroni, Michele Gragnanini, Rita Bottacchiari, Mattia Merlin, Giovanni Pulci, Francesco Marra

**Affiliations:** 1 INSTM Reference Laboratory for Engineering of Surface Treatments via Eudossiana 18, Department of Chemical Engineering, Materials, Environment, Sapienza University of Rome, 00184 Rome, Italy; rita.bottacchiari@uniroma1.it (R.B.); giovanni.pulci@uniroma1.it (G.P.); francesco.marra@uniroma1.it (F.M.); 2 Department of Engineering (DE), University of Ferrara, 44121 Ferrara, Italy; michele.gragnanini@unife.it (M.G.); mattia.merlin@unife.it (M.M.)

**Keywords:** electroless Ni-P coatings, nanocomposite coatings, ZrO_2_ and Al_2_O_3_ nanoparticles, dehydrogenation, thermal treatments, instrumented indentation, wear resistance

## Abstract

High phosphorus Ni-P coatings, both unreinforced and modified by the addition of alumina (Al_2_O_3_) and zirconia (ZrO_2_) nanoparticles, were manufactured by electroless deposition technique and heat-treated with different temperature and duration schedules. The effect of dehydrogenation (200 °C for 2 h) and its combination with crystallization heat treatment was studied in terms of microstructural changes and wear resistance. The amorphous structure of the coatings was not altered by the introduction of both Al_2_O_3_ and ZrO_2_ nanoparticles, and the addition of 1.5 g/L of ZrO_2_ yielded the highest microhardness due to better particles dispersion. Dehydrogenation improved hardness because of the early stages of grain growth; however, the greatest improvement in hardness (+120% compared to unreinforced Ni-P) was obtained after annealing at 400 °C for 1 h, because of the microprecipitation of the Ni_3_P crystalline phase induced by thermal treatment. No detectable differences in hardness and microstructure were detected when annealing at 400 °C for 1 h with or without prior dehydrogenation; however, the dehydrogenated coatings exhibited a lower Young’s modulus. ZrO_2_-reinforced coatings demonstrated improved wear resistance, and wear tests revealed that dehydrogenation is fundamental for lowering the coefficient of friction (−14%) and wear rate (−97%) when performed before annealing at 400 °C for 1 h. The analysis of the wear tracks showed that the non-dehydrogenated samples failed by complete coating delamination from the substrate, with abrasion identified as the predominant wear mechanism. Conversely, the dehydrogenated samples demonstrated better resistance due to the formation of a protective oxide layer, leading to an overall increase in the coating wear resistance.

## 1. Introduction

Electroless Ni-P coatings have been widely used in recent years for the wear and corrosion protection of industrial steel components [1,2,3,4]. The microstructure of the Ni-P alloy strongly depends on the P content: low P coatings (1–5 wt.%) are considered crystalline, medium P coatings (6–9 wt.%) have a nanocrystalline/mixed amorphous-crystalline structure and high P coatings (10–13 wt.%) are considered essentially amorphous [5,6,7,8,9]. In particular, electroless coatings containing high P amounts (HP) find extensive application in the energy production industry, where carbon steel components (such as compressor impellers, diaphragms, and nozzles) are characterized by a complex shaped geometry and cannot be easily coated by conventional methods like electrodeposition, physical vapor deposition (PVD), or thermal spray. Conversely, the electroless coating method, which is unaffected by the geometry of the component, ensures the deposition of uniform and conformal high phosphorous Ni-P coatings, providing good corrosion resistance in aggressive environments [4].

When amorphous HP coatings are placed in contact with corrosive media, preferential dissolution of Ni occurs, resulting in the formation of a P-rich outer layer that prevents further corrosion by chemical passivity [10,11]. In addition to this, the absence of grain boundaries limits the number of percolation paths for the penetration of corrosive agents, further increasing corrosion resistance [12,13]. On the other hand, the amorphous microstructure of HP confers inferior mechanical properties compared with medium P and low P alloys. This can be a considerable limitation considering that the operative environment typical of midstream and downstream Oil and Gas applications can lead to major degradation of components by unavoidable erosion and wear phenomena.

The most adopted strategies to improve the mechanical properties and confer good wear resistance to HP coatings, thus enhancing their applicability and durability, are the addition of functional second-phase hard particles and/or the adoption of post-fabrication heat treatments [5,14,15,16]. In recent years, several investigations have been performed on the manufacturing of particle-reinforced composite and nanocomposite Ni-P coatings [17,18,19,20,21,22]. Among all the proposed nanoparticles, the focus was often placed on the use of Al_2_O_3_ [23,24,25] and ZrO_2_ [26,27,28,29,30,31] since they are characterized by good mechanical resistance, high chemical stability, commercial availability, and high ζ-potential (ranging between 30 and 50 mV at pH 4.2), which is particularly important to guarantee their dispersion in the plating solution.

To the authors’ knowledge, the tribological properties of Ni-P coatings are not completely understood yet, especially when it comes to heat-treated nanocomposite coatings: only a few works have deeply investigated their wear behavior, specifically the wear mechanisms and the effect of different manufacturing procedures. Aslanyan et al. [32,33] investigated the effect of the addition of SiC particles on the wear behavior of Ni-P coatings both in unidirectional and bidirectional sliding. In the case of unidirectional sliding, they found that adding hard particles caused a lower coefficient of friction (COF) with respect to non-reinforced Ni-P, and abrasive wear was found to be the dominant wear mechanism. When bidirectional motion was considered, Ni-P and reinforced Ni-P showed similar wear behavior in terms of COF and wear rate, with oxidational wear found as the main wear mechanism. He et al. [34] focused on the effect of yttrium-stabilized zirconia with different yttrium contents on the corrosion and wear resistance of electroless Ni-P coatings. Balls made of Si_3_N_4_ were considered to focus all the worn material on the coatings in multidirectional motion. The reinforced coating showed the best tribological properties with negligible wear scars in comparison with traditional Ni-P. Abrasion and adhesive wear mechanisms were found to be the main causes of wear, with hardness acting as the main affecting factor.

Nonetheless, the evaluation of mechanical properties and wear resistance of Ni-P nanocomposites should be performed, taking into consideration that Ni-P deposition occurs along with H_2_ generation. Hydrogen is inevitably produced from a reaction parallel to the oxidation of the hypophosphite ion, according to Equations (1) and (2) [35]:

H_2_PO_2_^−^ + H_2_O → H_2_PO_3_^−^ + 2H^+^ + 2e^−^, (1)

2H^+^ + 2e^−^→ 2H_2_^−^, (2)

H_2_ bubbles form on the surface of the substrate and spontaneously take off when they reach a sufficient size. However, some hydrogen (0.08 ppm to 0.19 ppm for coating of about 25 µm thickness, according to [36]) remains trapped within the coating during its growth, causing considerable embrittlement that invariably degrades mechanical properties [37]. Moreover, the formation of small hydrogen cracks can also jeopardize the corrosion resistance [38,39]. It was demonstrated that trapped hydrogen can be effectively removed by performing dehydrogenation post-deposition heat treatment at 180–200 °C for at least 1 h [36,38,39,40,41]. Nonetheless, to the authors’ knowledge, an investigation into how dehydrogenation affects the wear resistance and wear mechanisms of Ni-P coatings (both standard and nanocomposite) is not available in the literature. Moreover, the requirement for post-deposition dehydrogenation in the case of successive heat treatments at higher temperatures has not been studied yet.

This work aims to fill this gap of knowledge by investigating the manufacturing and mechanical properties of Al_2_O_3_ and ZrO_2_ reinforced Ni-P nanocomposites subjected to different time/temperature schedules of heat treatment, focusing on how dehydrogenation affects the coating microstructure and wear mechanisms. For this purpose, different concentrations of Al_2_O_3_ and ZrO_2_ nanoparticles, with average particle sizes of 30–50 nm and 20–30 nm, respectively, were investigated, and the best nanocomposites were selected in terms of dispersion and distribution of particles and microhardness increase. Standard Ni-P and nanocomposite specimens were then heat-treated with three temperature-duration schedules: (i) 200 °C for 2 h, performed as dehydrogenation treatment; (ii) 400 °C for 1 h, to study the effect of annealing above the crystallization temperature; (iii) dehydrogenation and subsequent annealing at 400 °C for 1 h, to investigate how the combination of the two influences the coating properties. The aim was to uncover the changes in wear resistance along with nanoparticle introduction and microstructural changes by comparing the as-deposited Ni-P, both standard and nanocomposite, with those that underwent dehydrogenation and/or crystallization heat treatment. Particular attention was given to heat-treated nanocomposites, investigating the effect of annealing at 400 °C for 1 h with and without prior dehydrogenation, and aiming at understanding the involved wear mechanisms and defining manufacturing strategies that maximize tribological performance.

## 2. Materials and Methods

### 2.1. Coating Preparation

Disk-shaped specimens of F22 carbon steel (ASTM 182 standard [42]), with a 35 mm diameter and a 3 mm thickness, were used as substrates. Analytic grade chemicals purchased from Alfa Aesar (Thermo-Fisher Scientific, Waltham, MA, USA) were employed to prepare the plating solutions. Before deposition, all samples were sandblasted with corundum mesh 80 to achieve the same surface roughness (Ra = 3.877 ± 0.312 µm) and guarantee good adhesion, immersed for 1 min in an acidic solution containing HCl 37% diluted with 50 vol. % of water, to remove any superficial oxide, and eventually rinsed in deionized water. The solution for the deposition of Ni-P coatings with a high P content was prepared according to the procedure described in detail elsewhere [43]. The formulation of the plating solution and the deposition parameters are reported in Table 1. The amount of P in the coating was measured by Energy Dispersive X-Ray Spectroscopy (EDS): analysis was conducted on cross-sectioned specimens using a scanning window that comprised 80% of the coating thickness starting from the external interface (as similarly performed by [44,45]). Results indicated a P content equal to 11.44 ± 0.36 wt.%.

**Figure 1:**
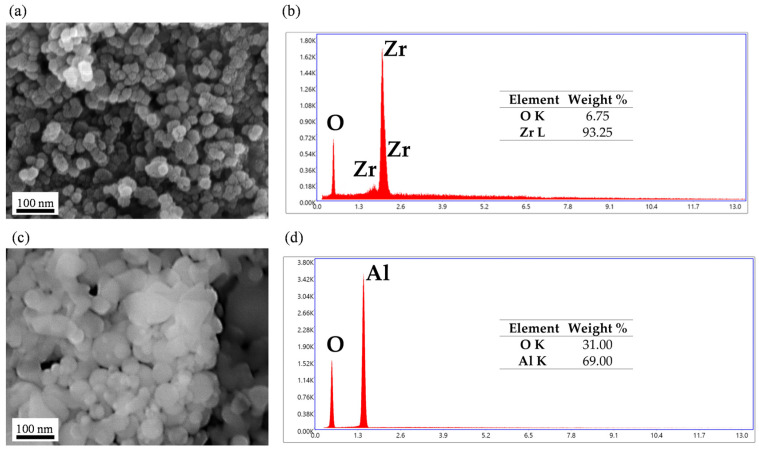
SEM micrographs and EDS analysis of as-purchased ZrO_2_ (**a**,**b**) and Al_2_O_3_ (**c**,**d**) nanoparticles.

**Figure 2:**
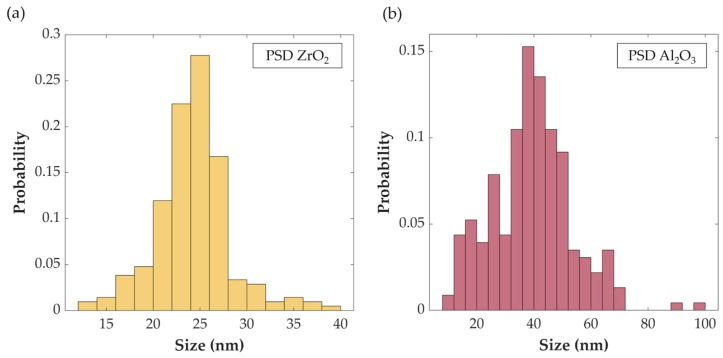
PSD of as-purchased ZrO_2_ (**a**) and Al_2_O_3_ (**b**) nanoparticles.

For the manufacturing of Ni-P nanocomposites, commercial ZrO_2_ and Al_2_O_3_ nanoparticles with average particle sizes of 20–30 nm and 30–50 nm, respectively, were purchased from Io-Li-Tec (Ionic Liquid Technologies GmbH, Heilbronn, Germany). SEM micrographs of the as-purchased ZrO_2_ and Al_2_O_3_ nanoparticles are shown in Figure 1a and c, respectively. The purity of the nanoparticles was confirmed by the EDS analysis reported in Figure 1b,d, in which only peaks attributed to Zr, O (in the case of zirconia), and Al and O (in the case of alumina) were identified. Particle size distributions (PSD) of the two sets of nanoparticles were estimated from over 200 measurements acquired by SEM micrographs and are shown in Figure 2a,b. The calculated mean size of nanoparticles was estimated at 24.2 nm for ZrO_2_ and 39.3 nm for Al_2_O_3_.

**Figure 3:**
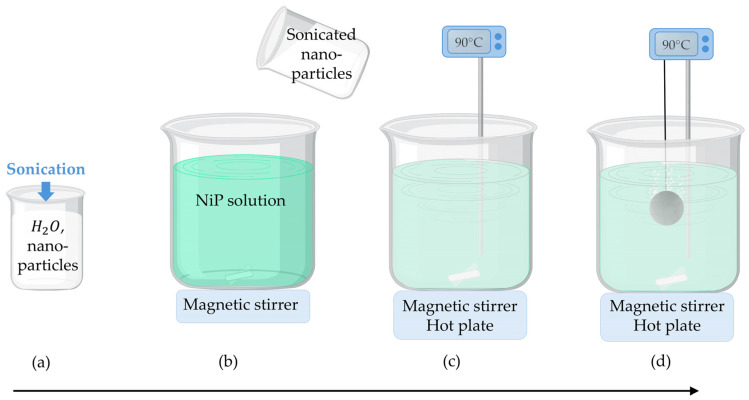
Deposition procedure of the Ni-P electroless nanocomposite coatings: (**a**) sonication of nanoparticles dispersed in water solution; (**b**) addition of sonicated nanoparticles to the Ni-P plating solution; (**c**) heating to 90 °C; (**d**) insertion of the sample in the plating solution.

ZrO_2_ and Al_2_O_3_ nanoparticles were selected because of their relatively high ζ-potential, which prevents their agglomeration into large clusters when dispersed in water solutions. For the manufacturing of nanocomposites, nanoparticles were added to aqueous solutions and sonicated with a Fisher Scientific 505 tip ultrasonic sonicator at 20% intensity for 10 min to promote dispersion before introducing them in the plating bath. Figure 3 summarizes the deposition procedure of the Ni-P electroless nanocomposite coatings. The final concentration of nanoparticles in the plating solution was varied between 0.5 g/L and 3 g/L for ZrO_2_ and between 1.5 g/L and 6 g/L for Al_2_O_3_. These quantities were defined to guarantee the same surface-to-volume ratio when adding particles of different sizes. Depositions were carried out at 90 °C for 120 min under continuous magnetic stirring and constant control over temperature.

### 2.2. Thermal Treatments

The manufactured coatings were heat-treated at 200 °C for 2 h and 400 °C for 1 h. The first time-duration schedule was selected since the growth process of Ni-P coatings invariably produces H_2_ along with Ni^2+^ reduction and H_2_PO_2_^−^ oxidation. To some extent, H_2_ may remain trapped within the coating, leading to considerable embrittlement of the material, thus reducing its wear resistance [38]. To remove embedded hydrogen, a thermal treatment at 200 °C for 2 h was necessary, and the properties of the dehydrogenated samples were compared with those of the as-deposited ones. Eventually, heat treatment at 400 °C for 1 h was performed to investigate the effect of crystallization on both dehydrogenated and non-dehydrogenated samples.

### 2.3. Coating Characterization

The coating thickness and nanocomposite microstructure were investigated by cross-sectional analysis using a Tescan Mira 3 (Tescan, Brno, Cechia) Field Emission-Scanning Electron Microscopy (FE-SEM) equipped with an Edax Octane Elect detector (Ametek Inc., Berwyn, PA, USA) for Energy Dispersive X-Ray Spectroscopy. Specimens for metallographic inspections were obtained by cutting the samples with a slow-speed linear precision saw: the cross-sections were mounted in epoxy resin (EpoThin 2, Buehler Ltd., Lake Bluff, IL, USA), ground with SiC papers (P400 to P1200 grit), and polished with water-based diamond suspensions (Buehler Ltd., Lake Bluff, IL, USA) up to 1 µm finishing.

Microstructure and crystalline phases were investigated by X-ray diffraction (XRD) analysis; XRD spectra were acquired using a Philips X’Pert diffractometer (PANalytical BV, Almelo, The Netherlands) operating at 40 kV and 40 mA with a CuKα1 radiation source. Acquisition parameters were: scan range of 20–80°, feed step of 0.02° and acquisition time of 2 s. Scherrer’s equation (Equation (3)) was used to calculate the crystallite size:

(3) $$D\;=\frac{0.94\lambda }{\beta \cos\left(\theta \right)}\;,$$

where λ is the wavelength of the radiation used, β is the peak broadening at half maximum intensity, and θ is the main peak position.

The crystallinity index was calculated from the XRD spectra as the ratio between the area under the crystalline peaks and the total area below the spectrum.

### 2.4. Microhardness and Instrumented Indentation Tests

Coatings microhardness was evaluated according to ASTM E384-11 [46] using a Leica VMHT (Leica GmbH, Wetzlar, Germany) testing machine equipped with a Vickers diamond indenter at 50 gf loading force with 15 s holding time. Measurements were acquired in cross-section to avoid any influence on surface morphology features. The results are reported in terms of the average value and standard deviation of at least twenty measurements for each coating type, with the distance between two indentations ≥25 μm.

Additional mechanical characterization was performed by instrumented indentation testing (ISO 14577-4:2016 [47]) using a Nanotest indenter (MicroMaterials Ltd., Wrexham, UK) equipped with a Berkovich tip. Depth vs. load hysteresis curves were recorded using a load-controlled method with a fixed time ramp and applying a maximum load of 250 mN. Tests were conducted with the following parameters: 0.5 mN initial load, 20 s loading/unloading time, and 10 s dwell time at maximum load. Given the lower test load and punctual contact guaranteed by the use of a Berkovich tip, the hardness (H) and Young’s modulus (E) are calculated, minimizing the influence of coating defects and operator bias (which can be more prominent in Vickers testing, especially at low loads). Tests were conducted on mirror-polished surfaces to avoid the influence of roughness. Polishing was performed using P1200 SiC paper and water-based diamond suspensions at a very low load to minimize the hardening effect. At least 20 indentation cycles were performed for each sample. Hardness was calculated according to Equation (4) [48]:

(4) $$H\;=\frac{P_{\max}}{A}\;,$$

where P_max_ is the maximum load and A is the contact area under that load. Young Yung’s modulus of the coatings was derived from the reduced Young’s modulus (E_r_), according to Equation (5) [48]:

(5) $$\frac{1}{E_{r}}=\frac{\left(1-{\;\nu }^{2}\right)}{E}+\frac{\left(1-{\;\nu }_{i}^{2}\right)}{E_{i}}\;,$$

where ν is the Poisson ratio of the sample, considered equal to 0.31 [49,50,51], and ν_i_ and E_i_ are the Poisson ratio and the Young’s modulus of the indenter, respectively. In the case of the Berkovic three-sided pyramidal indenter, the Poisson ratio is considered 0.07, and the elastic modulus is equal to 1141 GPa [52].

The analysis of both the surface morphology and roughness (Ra) of the unreinforced Ni-P and nanocomposite coatings before and after heat treatments was conducted using a Taylor-Hobson optical non-contact profilometer (Tayor-Hobson, Leicester, UK). The average values of Ra and standard deviations were calculated using MountainsMap software (v10.1, Digital Surf, Besançon, France) according to ISO 21920-2:2021 [53].

### 2.5. Wear Tests

Ball-on-disk tribological tests were conducted in unidirectional motion at a constant sliding speed equal to 0.05 m/s and a 30 N normal load. Tests were performed on disks coated with standard Ni-P coatings and on the best nanocomposites selected in terms of particle distribution and microhardness. All samples were tested in the as-coated state and after dehydrogenation (annealing at 200 °C for 2 h). Moreover, to unveil the necessity of dehydrogenation to improve the wear resistance of crystallized Ni-P, nanocomposites were also tested after heat treatment at 400 °C for 1 h with and without previous dehydrogenation. All the samples selected for the instrumented indentation tests and wear tests are summarized in Table 2.

Spheres made of Al_2_O_3_ with a 6 mm diameter were selected as counterparts for their high hardness (≥1600 HV) to study the tribological behavior of the coatings. Three replicates were performed on the same surface of each sample at three different diameters until the same number of cycles, equal to 1200, was reached. The number of cycles was defined according to the literature. Okonkwo et al., obtained consistent friction and wear results using the same methodology [54].

The wear scars of the disks were measured using a Taylor-Hobson 3-D optical non-contact profilometer (Tayor-Hobson, Leicester, UK) to determine the transversal area of each wear track; subsequently, the volume loss was calculated using MountainsMap software (v10.1, Digital Surf, Besançon, France). The specific wear rate (WR) was calculated and considered as a parameter for comparison.

## 3. Results and Discussion

### 3.1. Microhardness and Microstructural Characterization of Coatings

The thickness of the coatings was measured from cross-section SEM micrographs. Ni-P samples exhibited an average thickness of 50.7 ± 2.8 µm, while the evaluated thicknesses of the Ni-P + 3 g/L Al_2_O_3_ and the Ni-P + 1.5 g/L ZrO_2_ were 52.3 ± 2.8 µm and 58.0 ± 2.1 µm, respectively.

**Figure 4:**
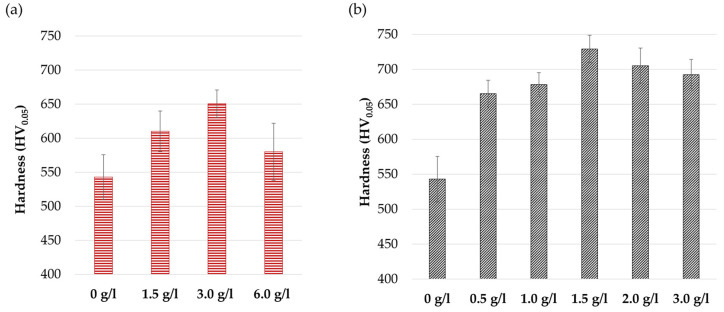
Coatings microhardness as a function of Al_2_O_3_ (**a**) and ZrO_2_ (**b**) nanoparticle concentration in the plating solution.

The coating microhardness as a function of the nanoparticle concentration in the plating solution is shown in Figure 4a for Al_2_O_3_ nanoparticles and Figure 4b for ZrO_2_ nanoparticles, considering the standard Ni-P coatings as references (indicated as 0 g/L). It can be noted that in both cases, the nanocomposite coatings exhibit higher microhardness compared with the particle-free coatings, highlighting an effective dispersion-hardening effect. Indeed, nanoparticles act as obstacles to dislocation motion, increasing the energy required for their propagation in a ductile matrix, according to the Orowan strengthening mechanism [55,56]. The microhardness increases with increasing concentration of nanoparticles in the plating solution. However, this increase reaches a maximum value at a certain threshold concentration, after which a slight decrease is observed. This behavior can be ascribed to the agglomeration phenomenon: the higher the concentration of nanoparticles in the solution, the lower the mean distance between the particles, and the higher their probability to agglomerate. The embedding of agglomerates within the coating decreases the Orowan strengthening efficacy, which strongly depends on the nanoparticle size and their dispersion and distribution. Moreover, the incorporation of large agglomerates may occur along with the formation of micro-voids within and around the nanoparticle clusters [57], invariably degrading the structural integrity and mechanical properties. The maximum hardness is obtained with the introduction of 3 g/L of Al_2_O_3_ nanoparticles and 1.5 g/L of ZrO_2_ nanoparticles; the highest increase in hardness is reached with the addition of ZrO_2_ nanoparticles.

**Figure 5:**
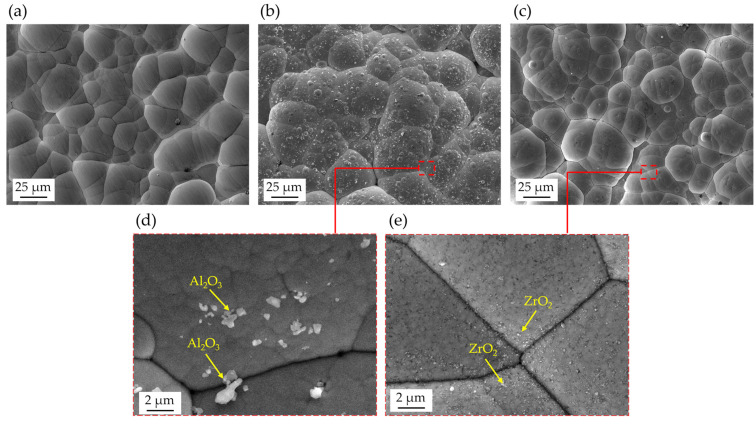
Top view SEM micrographs showing morphology of (**a**) standard Ni-P, (**b**,**d**) nanocomposite reinforced with 3 g/L of Al_2_O_3,_ and (**c**,**e**) nanocomposite reinforced with 1.5 g/L of ZrO_2_. Gray phase: Ni-P. Bright phases on the surface indicated by yellow arrows are partially embedded nanoparticles.

Top view SEM micrographs of standard Ni-P, nanocomposite reinforced with 3 g/L of Al_2_O_3,_ and nanocomposite reinforced with 1.5 g/L of ZrO_2_ are reported in Figure 5a, b, and c, respectively. All coatings exhibit a cauliflower-like morphology typical of electroless Ni-P coatings, which is attributed to the deposition mechanism by nucleation, growth, and coalescence phenomena. Nanocomposite coatings are characterized by a less regular distribution of nodules, which appear refined and are more variable in size compared to standard Ni-P. This can be explained by the presence of nanoparticles, which are incorporated into the coating during deposition and might limit the lateral growth of single nodules [26,58,59]. In addition, a small amount of partially embedded nanoparticles can be observed on the surface of these coatings, as shown in the higher magnification micrographs in Figure 5d,e.

**Figure 6:**
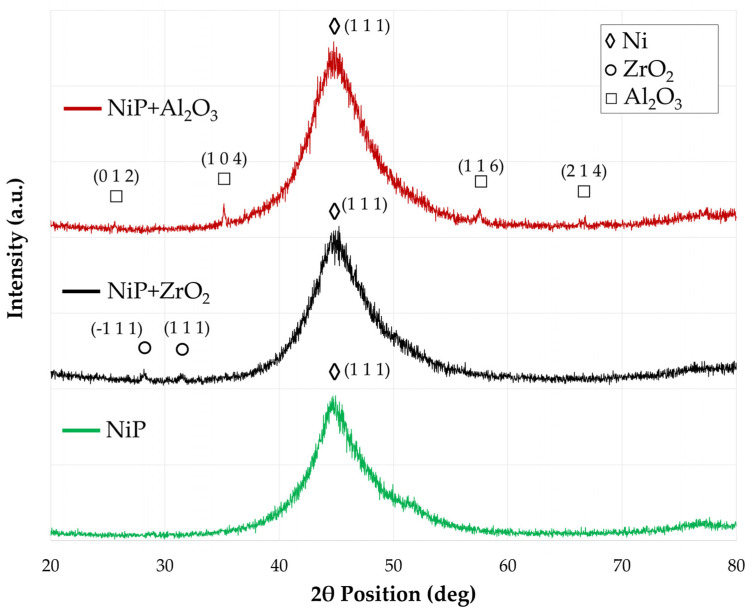
XRD spectra of unreinforced Ni-P coatings (green line), nanocomposite reinforced with 1.5 g/L of ZrO_2_ (black line), and nanocomposite reinforced with 3.0 g/L of Al_2_O_3_ (red line) in the as-coated condition.

The effect of nanoparticle introduction on the microstructure of high P electroless Ni-P coatings was investigated by XRD analysis. Spectra of unreinforced Ni-P, nanocomposite reinforced with 1.5 g/L of ZrO_2,_ and nanocomposite reinforced with 3.0 g/L of Al_2_O_3_ in the as-coated condition are reported in Figure 6. All coatings exhibit the typical amorphous profile of Ni-P coatings with high P content (11.44 ± 0.36 wt.%) [9,60,61], with a single broad peak of Ni, attributed to Ni(111) (JCPDS 70-0989), located at 35–55° angular position. P atoms located at interstitial positions distort the nickel lattice to the extent that long-range order is lost, and the matrix can be considered amorphous. The only difference between the three spectra is the presence of peaks belonging to ZrO_2_ (JCPDS 78-0047) and Al_2_O_3_ (JCPDS 46-1212) particles in the case of the nanocomposites, demonstrating the effective incorporation of nanoparticles without altering the microstructure of the matrix.

**Figure 7:**
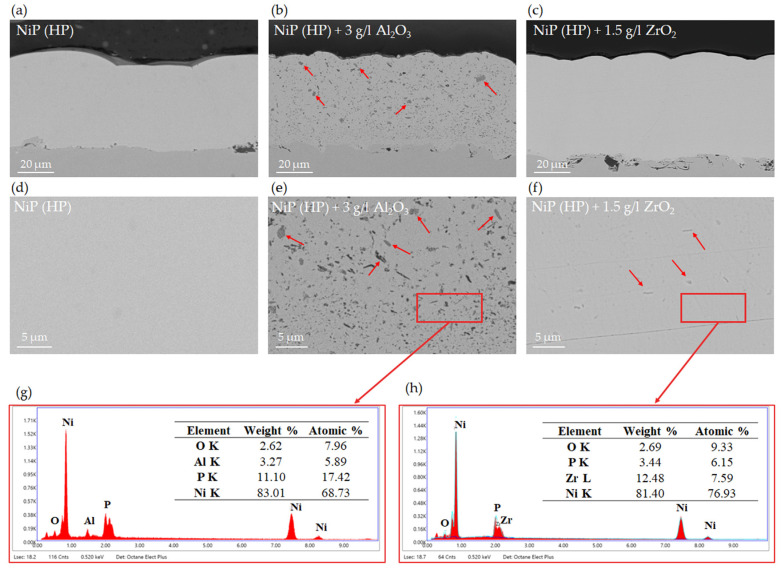
Cross-section backscattered electron SEM micrographs of standard Ni-P coatings (**a**,**d**), nanocomposites reinforced with 3 g/L of Al_2_O_3_ (**b**,**e**), nanocomposites reinforced with 1.5 g/L of ZrO_2_ (**c**,**f**) and related EDS analysis (**g**,**h**). The arrows (**b**,**e**,**f**) highlight the agglomerates of the nanoparticles.

To better understand the reason for the higher hardness measured in ZrO_2_-modified coatings, SEM analyses were performed on the cross-sectioned specimens in the backscattered electron (BSE) imaging mode. Figure 7 shows representative micrographs of standard Ni-P (Figure 7a,d), Ni-P reinforced with 3 g/L of Al_2_O_3_ (Figure 7b,e) and 1.5 g/L of ZrO_2_ (Figure 7c,f). It is worth noting that all the coatings are dense and crack-free. As widely reported in the literature [16,31], the overall increase in microhardness depends on both the amount of inserted hard reinforcement and the dispersion of ceramic nanoparticles. The Al_2_O_3_ nanocomposite exhibits a non-uniform dispersion of particles along the coating thickness, and large agglomerates are present in the external half of the coating (as indicated by red arrows). Conversely, ZrO_2_ nanoparticles, despite being less visible in the BSE-SEM micrographs due to the lower compositional contrast, appear well-dispersed and well-distributed, with only some more visible agglomerates that can be identified in Figure 7f. The presence of these agglomerates is usually a consequence of agglomeration processes in the plating solution [31] and appears more likely to occur in the case of Al_2_O_3_ dispersions, despite the comparable ζ-potential of the two investigated nanoparticles at the pH value of deposition (equal to 4.2) [62]. Moreover, the nominal smaller size of ZrO_2_ nanoparticles, coupled with their lower agglomeration degree, might lead to more effective Orowan strengthening mechanisms and, therefore, better hardening.

**Figure 8:**
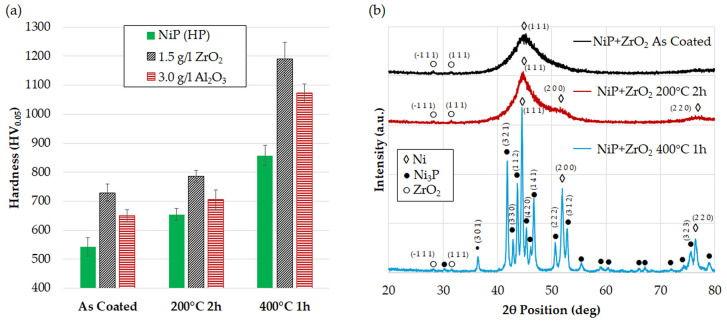
(**a**) Microhardness of particle-free and nanocomposite coatings (**a**) and microstructural evolution (**b**) after thermal treatment.

The results of the microhardness tests performed on the coatings after thermal treatment at 200 °C for 2 h and 400 °C for 1 h are shown in Figure 8a, and the corresponding XRD microstructural changes are presented in Figure 8b. As previously demonstrated, no microstructural changes of the Ni-P matrix are observed after the addition of ZrO_2_ and Al_2_O_3_ nanoparticles: the only difference lies in the appearance of characteristic peaks that can be attributed to the nanoparticles; therefore, only ZrO_2_ nanocomposite spectra are reported for simplicity. A slight hardness increase is observed after the dehydrogenation treatment, mainly as a consequence of initial grain nucleation and growth. Compared to the XRD spectra in the as-deposited condition, the spectrum acquired after treatment at 200 °C for 2 h exhibits a better-defined and less broad Ni(111) peak, and the initial appearance of Ni(200) and Ni(220) peaks at 52.03° and 76.99°. These considerations indicate that some limited grain growth occurs after dehydrogenation, which also causes an increase in microhardness according to the inverse Hall-Patch mechanism [63]. The hardness increase is considerably higher (+20%) for the particle-free coatings compared with the nanocomposites (+8.0% for the ZrO_2_ nanocomposite and +8.5% for the Al_2_O nanocomposite), suggesting that nanoparticle incorporation might retard grain growth and hamper its hardening effect. Similar results were reported by Dhakal et al. [64], who suggested that the presence of nanoparticles induces strain within the lattice, which, in turn, constrains grain growth. Consistent findings have often been reported in the literature [22,58,65,66]. Further hardening occurs after heat treatment at 400 °C for 1 h as a consequence of Ni crystallization and precipitation of nanometric Ni_3_P hard phases (JCPDS–34-0501), which provides an effective precipitation strengthening mechanism [66,67,68]. The crystal sizes evaluated by Scherrer’s equation and the lattice parameters of the phases detected by XRD analysis are listed in Table 3.

No obvious differences in microstructure and microhardness are observed when heat treatment at 400 °C for 1 h is performed with and without prior dehydrogenation; the grain size of Ni remains 50.7 nm in both cases, and that of Ni_3_P precipitates decreases slightly from 56.5 nm to 50.2 nm. Indeed, the microstructural changes that occur when annealing above the crystallization are so massive that the small modifications observed after dehydrogenation become negligible. Nonetheless, the hydrogen embrittlement phenomenon can play a role when considering coating resistance. To uncover this aspect, standard Ni-P, nanocomposites, and heat-treated coatings were subjected to instrumented indentation tests and tribological tests.

### 3.2. Instrumented Indentation Tests

Instrumented indentation tests were conducted on standard coatings and on nanocomposites reinforced with 1.5 g/L of ZrO_2_ due to their higher microhardness and better dispersion of the reinforcing phase. From now on, samples will be referred to as Ni-P for the unreinforced Ni-P, Ni-P/ZrO_2_ for the nanocomposites, and Ni-P/ZrO_2_/TT400 °C 1 h for the ZrO_2_ reinforced Ni-P nanocomposite annealed at 400 °C for 1 h. Performing dehydrogenation at 200 °C for 2 h will be specified in each specific case.

**Figure 9:**
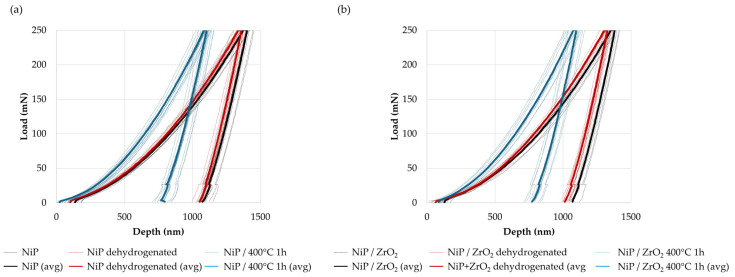
Load-displacement curves from the instrumented indentation of standard Ni-P (**a**) and ZrO_2_ reinforced nanocomposites (**b**) in the as-deposited state after dehydrogenation and after heat treatment at 400 °C for 1 h (avg stands for average).

**Figure 10:**
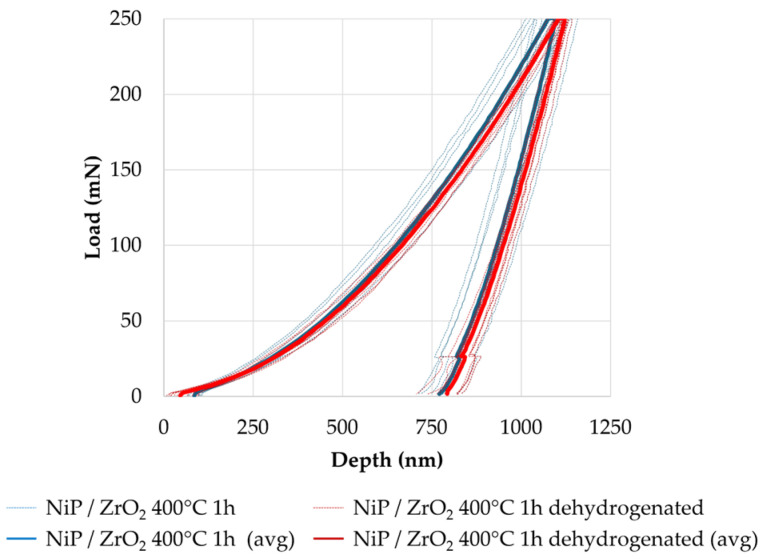
Load-displacement curves from the instrumented indentation of ZrO_2_ nanocomposites heat-treated at 400 °C for 1 h with (red line) and without (blue line) prior dehydrogenation (avg stands for average).

The load-displacement curves of standard Ni-P and ZrO_2_ nanocomposites recorded by instrumented indentation tests are reported in Figure 9 and Figure 10. Dotted lines represent all curves calculated for each test condition, while solid lines represent the average. The hardness, Young’s Modulus, and H/E ratio for each set of samples, calculated at 250 mN maximum load, are listed in Table 4. The results clearly demonstrate that both thermal treatment and the addition of hard ZrO_2_ nanoparticles lead to an improvement in the coating hardness (consistent with the Vickers microhardness test results) and to an increase in the elastic modulus. The higher hardness of nanocomposites, compared with particle-free coatings, is ascribed to an effective dispersion-hardening according to the Orowan mechanism [55,56]. In addition, the stiffening effect may result from the synergistic effect of two components: (i) the microstrain introduced by the dispersion of nanoparticles, which can alter the lattice parameters of Ni and Ni_3_P grains; and (ii) microstructural modifications, including Ni_3_P precipitation, induced by heat treatments [15,22,64]. It is worth noting that a slight reduction in the elastic modulus is registered when the heat treatment at 400 °C for 1 h is preceded by dehydrogenation, despite the comparable hardness value, and the results are characterized by a lower dispersion of data. This suggests that dehydrogenation plays a role in the relaxation of deposition stresses; this effect, which is negligible before crystallization, becomes significant when additional strain components are introduced by heat treatment at 400 °C.

A quantitative representation of how the combination of hardness and stiffness can represent the mechanical resistance of coatings is provided by the H/E ratio. According to the work by Leyland and Matthews [69], the H/E parameter can be effective for the preliminary assessment of the wear resistance of coatings (a higher H/E ratio indicates higher wear resistance). The results in Table 4 show that H/E increases with nanoparticle incorporation in all cases. Moreover, it also increases after dehydrogenation in both the case of standard Ni-P and nanocomposite coatings, confirming the importance of this post-deposition treatment to guarantee better protective properties of the coatings.

### 3.3. Wear Tests

Wear tests were performed to further investigate the effect of nanoparticle addition and heat treatments on the tribological behavior of the coatings. Before conducting the wear tests, the arithmetic average surface roughness (Ra) of the samples was evaluated, and the results are reported in Table 5. Nanocomposite coatings exhibit lower Ra values than standard Ni-P; this phenomenon is related to the nodular refinement observed as a consequence of nanoparticle introduction (Figure 5), and similar results have often been reported in the literature [21,26,34]. A further reduction in Ra is obtained after heat treatment at 400 °C for 1 h, consistent with the additional nodular refinement that is known to occur after the crystallization of the Ni-P matrix [68]. No significant differences are observed when t dehydrogenation heat treatment is performed.

**Figure 11:**
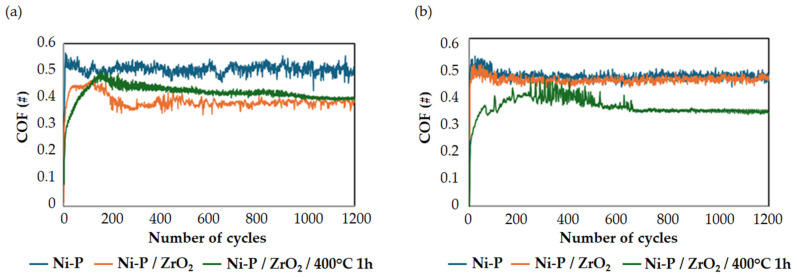
COF evolution against 1200 cycles of sliding for (**a**) untreated samples and (**b**) dehydrogenated samples. # stands for dimensionless number.

The COF evolution against 1200 cycles for each considered sample, calculated as the average of the three replicas, is shown in Figure 11. In the case of the non-dehydrogenated samples (see Figure 11a), Ni-P/ZrO_2_ showed the lowest COF, which was slowly reached in the final stage of the test; the same trend was also observed in the case of Ni-P/ZrO_2_/400 °C 1 h. The as-coated Ni-P coating showed the highest value of COF and the highest data fluctuation. The same dispersion of COF data was observed for Ni-P/ZrO_2,_ and this behavior can be attributed to the onset of stick-slip mechanisms [70].

Conversely, the dehydrogenated samples exhibited an overall smoother COF evolution (Figure 11b); no noteworthy differences were detected between Ni-P and Ni-P/ZrO_2_, indicating that the reinforcement does not play a main role in the COF evolution. The COF of Ni-P/ZrO_2_ increased after dehydrogenation, while the heat-treated Ni-P/ZrO_2_/400 °C 1 h showed a lower steady-state COF after a noisier initial transient.

The average values of the COF and standard deviation for each sample are listed in Table 6. No substantial differences were detected for the Ni-P samples despite the lower average COF value after dehydrogenation. Their COF values also confirmed the difference between the untreated and dehydrogenated Ni-P/ZrO_2_ samples, which could be related to the absence of the transition that occurred between 100 and 200 cycles for the untreated Ni-P/ZrO_2_ samples. The Ni-P/ZrO_2_/400 °C 1 h samples seemed to be the ones most influenced by the dehydrogenation treatment, with a significant drop in the COF average values. Both the as-coated and dehydrogenated coatings presented a COF average value sensibly lower than the results reported by Gay et al. [71] while studying the wear resistance of ZrO_2_ reinforced Ni-P coatings in unidirectional friction tests with a 2 N applied load and 100Cr6 steel balls as counterparts.

**Figure 12:**
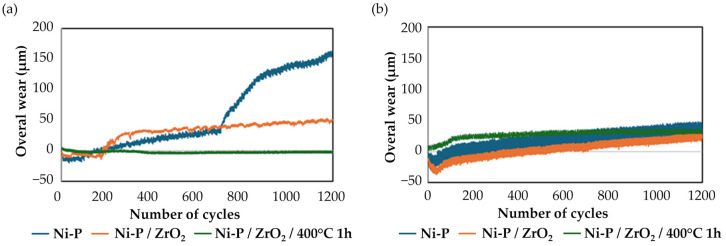
Overall wear of the system against the number of cycles of sliding for (**a**) non-dehydrogenated and (**b**) dehydrogenated samples.

To better understand the changes in the COF behavior, the overall wear of the tribo-system, measured as the vertical displacement of the ball during the test, was considered. It is calculated by an integrated LVDT (Linear Variable Displacement Transducer) sensor, which jointly considers the wear of the specimen and that of the counterpart. The overall wear evolution against the number of cycles is shown in Figure 12 for both the untreated and dehydrogenated samples. In the case of the untreated samples (Figure 12a), the results confirmed that for both Ni-P and Ni-P/ZrO_2,_ a transition occurred before the end of the test. The overall wear signal started to increase with a high slope after 700 cycles for HP and with a lower slope for Ni-P/ZrO_2_ just before 200 cycles: this behavior can be associated with failure by spallation and adhesion-related issues [66] since internal stresses can make non-dehydrogenated coatings more susceptible to collapse under external pressure. Considering the Ni-P/ZrO_2_/400 °C 1 h condition, no significant slope variations were detected during sliding. In the case of the dehydrogenated samples (Figure 12b), no transition occurred for any of the samples; the Ni-P and Ni-P/ZrO_2_ coatings exhibited a very similar overall wear evolution, while the dehydrogenated Ni-P/ZrO_2_/400 °C 1 h seemed to be worn in the very beginning of the sliding until it reached a steady-state regime with quasi-zero wear.

**Figure 13:**
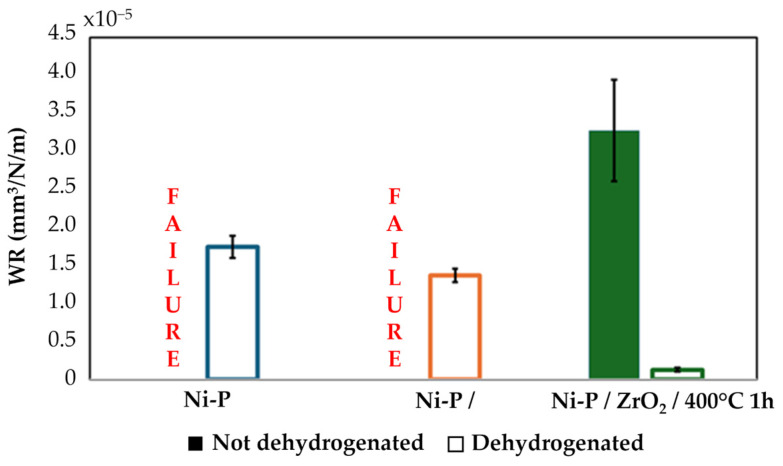
WR values for all the NiP coatings under not dehydrogenated and dehydrogenated conditions.

To better compare the wear behaviors of the different coatings, the WR was calculated. All the WRs are graphically reported in Figure 13 for both the untreated and dehydrogenated samples. Based on the obtained results, dehydrogenation improved wear behavior lowering all the WR values. In the case of both untreated Ni-P and Ni-P/ZrO_2_ samples, the calculation could not be performed because of the failure of the coatings: this also explains the transitions that were noticed for both the COF values and overall wear evolutions in Figure 11a and Figure 12a, respectively. After dehydrogenation, all the samples overcame the sliding test, and the WR was calculated for each sample. It can be observed that the nanoparticle reinforcement improved the wear resistance of standard Ni-P, and that the best performance was obtained in the heat-treated condition. Biswas et al. [72] obtained a similar trend testing high phosphorus Ni-P coatings in as-coated conditions and after different heat treatments. The authors observed that annealing performed at 400 °C for 1 h led to the lowest WR, testing the coatings in unidirectional motion at a sliding speed of 0.157 m/s and applying a load of 20 N. Gadhari and Sahoo [73] also demonstrated that annealing at 400 °C led to the lowest WR testing Ni-P-Al_2_O_3_ nanocomposites coatings annealed following different time and temperature combinations in unidirectional sliding tests under the effect of a 50 N normal load and using Al_2_O_3_ spheres as counterpart. The results are also in agreement with the H/E ratio computed for each considered layer, as the WR decreased with increasing H/E ratio [69].

**Figure 14:**
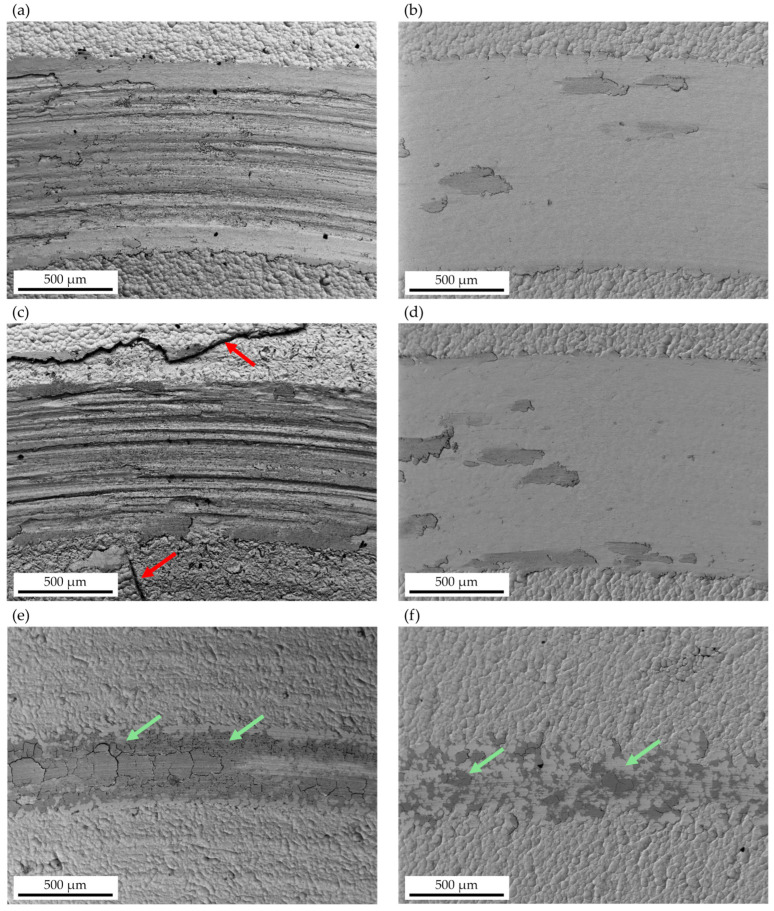
SEM micrographs of the wear tracks for Ni-P (**a**), Ni-P/ZrO_2_ (**c**), and Ni-P/ZrO_2_/400 °C 1 h (**e**) without prior dehydrogenation and for Ni-P (**b**), Ni-P/ZrO_2_ (**d**), and Ni-P/ZrO_2_/400 °C 1 h (**f**) with prior dehydrogenation. Red arrows indicate surface cracks (**c**), while green arrows indicate a dark layer of compacted wear debris (**e**,**f**).

To comprehensively understand the wear behaviors of the different Ni-P coatings, the worn surfaces of each sample were investigated through SEM imaging. Representative micrograph images of the wear tracks are shown in Figure 14. Two different wear mechanisms were detected for the as-deposited coatings (Figure 14a,c,e). Large wear tracks were observed on Ni-P and Ni-P/ZrO_2,_ and in both cases, deep and wide grooves were visible along the wear track in the sliding direction, suggesting abrasion as the predominant wear mechanism. Wide cracks can be observed on the surface of the Ni-P/ZrO_2_ coating (red arrows in Figure 14c) on both sides of the wear track, suggesting failure of the coatings. These findings are consistent with the WR values reported in Figure 12. Ni-P/ZrO_2_/400 °C 1 h sample showed improved wear resistance compared with Ni-P/ZrO_2_, as the width of the wear track decreased and a dark layer of well-compacted debris appeared widely distributed over the entire wear track. Conversely, a completely different behavior was observed for the dehydrogenated samples (Figure 14b,d,f), as indicated by the morphology of the wear track and confirmed by the improved wear resistance: both dehydrogenated Ni-P and Ni-P/ZrO_2_ coatings did not exhibit grooves on the wear scars, indicating that abrasion did not occur as the predominant wear mechanism; discontinuous dark and plastically deformed debris were detected across the wear track, and spread over it. The same wear mechanisms were also observed for dehydrogenated Ni-P/ZrO_2_/400 °C for 1 h, and the same dark layer of well-compacted debris was observed over the entire wear track (see the green arrows in Figure 14e,f). The different observed wear mechanisms suggest that dehydrogenation heat treatment improves wear behavior by reducing the hydrogen embrittlement phenomenon, which can be the cause of lower resistance and failure by delamination and by increasing the coating hardness.

**Figure 15:**
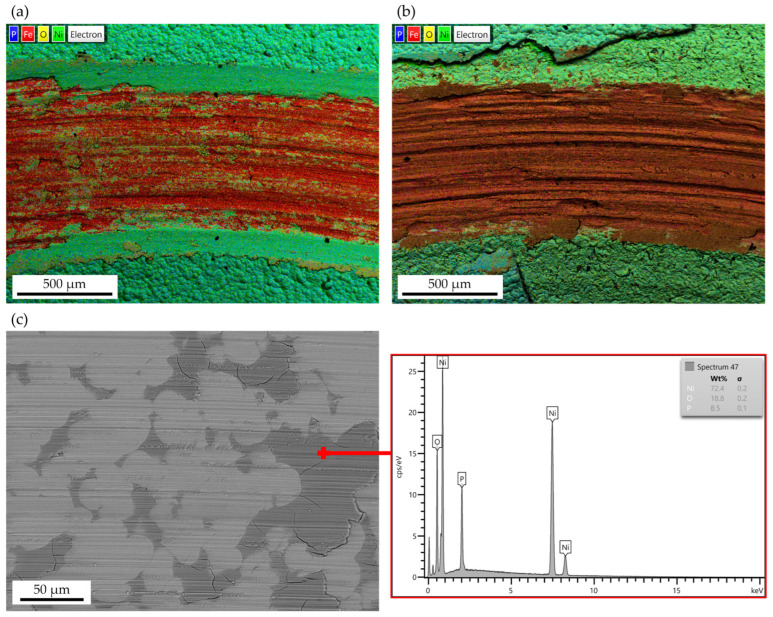
(**a**) EDS map of the as-deposited Ni-P wear track; (**b**) EDS map of the as-deposited Ni-P/ZrO_2_ wear track; (**c**) EDS analysis of a typical wear track of the dehydrogenated samples, highlighting the formation of an oxide film.

Semiquantitative EDS analyses were performed to confirm the main wear mechanisms, and the results are shown in Figure 15. The diffused presence of iron (Fe) was detected across the wear tracks of non-dehydrogenated Ni-P and Ni-P/ZrO_2_ (Figure 15a,b), confirming that in both cases, complete failure occurred through delamination of the coating from the steel substrate. Conversely, when dehydrogenation was performed, thinner scratches appeared longitudinally to the sliding direction, as reported in the higher magnification micrograph in Figure 15c, and a protective oxide layer was observed across the wear track of all Ni-P coatings. The same oxidative phenomenon was also observed by Aslayan et al. [33], who investigated SiC-reinforced Ni-P composites in unidirectional sliding and attributed the temporary protective effect to the formation of an oxide layer. These findings are consistent with the WR results presented in Figure 13.

As observed by León-Patiño et al. [14], oxidation phenomena were also found to have a protective effect on the wear resistance of Ni-P coatings reinforced with Al_2_O_3_ nanoparticles. The authors tested non-reinforced high-P Ni-P coatings and found that debris from the matrix reacts with the environment and oxidizes; then, they are partially compacted by the counterpart and form a thin layer that protects the coating from severe wear. However, compared to the present study, higher WR values were obtained despite the lower load applied, confirming the better properties achieved with the addition of ZrO_2_ nanoparticles. Similar results regarding the formation of the thin protective oxide layer shown in Figure 15c were reported by He et al. [34].

## 4. Conclusions

In the present work, the synergistic reinforcement effect of nanoparticle addition and heat treatment on the mechanical and tribological behavior of electroless Ni-P coatings with a high P content was investigated. It was demonstrated that the introduction of ZrO_2_ nanoparticles led to a more effective microhardness increase compared with introduction of Al_2_O_3_. The overall hardness increase by ZrO_2_ addition is higher than 35% compared with unreinforced Ni-P. Nanoparticles do not alter the microstructure of as-coated Ni-P samples and thermal treatments are required to induce microstructural changes: (i) dehydrogenation heat treatment at 200 °C for 2 h leads to initial Ni grain growth (without Ni_3_P precipitation) and +50% microhardness increase for the nanocomposites compared to standard as-deposited Ni-P; (ii) annealing at 400 °C for 1 h leads to crystallization and Ni_3_P precipitation, with hardness increase up to 130%.

The study also highlighted the critical role of dehydrogenation heat treatment in enhancing the coatings’ performance. Dehydrogenation at 200 °C for 2 h mitigated the negative effects of hydrogen embrittlement and provided relaxation of deposition stresses, significantly improving hardness, Young’s modulus, and wear resistance, especially when performed prior to crystallization heat treatment at 400 °C for 1h.

Wear tests revealed that dehydrogenation is crucial for reducing the coefficient of friction (COF) and wear rate (WR). Without dehydrogenation, both the standard and nanocomposite Ni-P coatings failed due to the complete delamination of the coating from the steel substrate, and abrasion was the predominant wear mechanism. Conversely, the dehydrogenated samples demonstrated better resistance due to the formation of a protective oxide layer. When dehydrogenation was followed by annealing at 400 °C for 1 h, further improvements were observed due to the crystallization of Ni and the precipitation of hard Ni_3_P phases, which added additional strengthening to the coatings.

In conclusion, this study demonstrates that Ni-P coatings reinforced with ZrO_2_ nanoparticles subjected to appropriate dehydrogenation and crystallization heat treatments offer superior hardness and wear resistance. These findings provide valuable insights into the optimization of Ni-P nanocomposite coatings for industrial applications.

## Figures and Tables

**Table 1:** Formulation of electroless Ni-P plating solution and deposition parameters [43].

Compound	Concentration (g/L)
NaH_2_PO_2_ ∙ H_2_O	110
C_2_H_3_NaO_2_	20
C_6_H_8_O_7_	9
NiSO_4_ ∙ 6H_2_O	25
Thiourea	8.5 ppm ^a^
pH	4.2
Temperature	90 °C

^a^ Added from 1M water solution.

**Table 2:** Summary of samples subjected to wear tests.

Specimen	Thermal Treatments
Standard Ni-P	None
Standard Ni-P	Dehydrogenated
Ni-P nanocomposite	None
Ni-P nanocomposite	Dehydrogenated
Ni-P nanocomposite	400 °C 1 h
Ni-P nanocomposite	Dehydrogenated + 400 °C 1 h

**Table 3:** Summary of grain size and lattice parameters evaluated from the XRD results.

Sample	Phase	Space Group	Lattice Constant (Å)	Ni Grain Size (nm)	Crystallinity Index (%)
**Ni-P**	Ni	Fm-3m	3.5238	1.96	40
**Ni-P/Al_2_O_3_**	Ni	Fm-3m	3.5238	1.83	49
	Al_2_O_3_	R-3c	a, b = 4.7587 c = 12.9929	n.a.	
**Ni-P/ZrO_2_**	Ni	Fm-3m	3.5238	1.85	42
	ZrO_2_	P21/c	a = 5.1507 b = 5.2028 c = 5.3156	n.a.	
**Ni-P/ZrO_2_/200 °C 2 h**	Ni	Fm-3m	3.5238	2.58	58
	ZrO_2_	P21/c	a = 5.1507 b = 5.2028 c = 5.3156	n.a.	
**Ni-P/ZrO_2_/400 °C 1 h**	Ni	Fm-3m	3.5238	50.7	77
	Ni_3_P	I-4	a, b = 8.9520 c = 4.3880	56.5	
	ZrO_2_	P21/c	a = 5.1507 b = 5.2028 c = 5.3156	n.a.	

**Table 4:** Hardness (H), Young’s modulus (E), and H/E ratio of standard Ni-P coatings and ZrO_2_-reinforced nanocomposites in the as-deposited state and after different heat treatment conditions, measured by instrumented indentation tests.

Specimen	Hardness (GPa)	Young’s Modulus (GPa)	H/E Ratio
Ni-P	6.26 ± 0.24	138.9 ± 5.4	0.0451 ± 0.0025
Ni-P dehydrogenated	6.93 ± 0.26	144.7 ± 6.9	0.0479 ± 0.0029
Ni-P/400 °C 1 h	9.37 ± 0.47	181.6 ± 13.3	0.0516 ± 0.0046
Ni-P/400 °C 1 h dehydrogenated	9.39 ± 0.31	174.4 ± 9.6	0.0538 ± 0.0035
Ni-P/ZrO_2_	6.56 ± 0.30	142.6 ± 5.3	0.0460 ± 0.0027
Ni-P/ZrO_2_ dehydrogenated	7.10 ± 0.21	149.4 ± 13.3	0.0482 ± 0.0045
Ni-P/ZrO_2_/400 °C 1 h	10.86 ± 0.87	198.0 ± 10.3	0.0548 ± 0.0052
Ni-P/ZrO_2_/400 °C 1 h dehydrogenated	10.78 ± 0.52	189.5 ± 7.1	0.0569 ± 0.0035

**Table 5:** Ra values of the substrate before the coating process and of the samples subjected to wear tests.

	Substrate (SandBlasted)	Ni-P	Ni-P/ZrO_2_	Ni-P/ZrO_2_ Dehydrogenated	Ni-P/ZrO_2_/400 °C 1 h	Ni-P/ZrO_2_/400 °C 1 h Dehydrogenated
Ra (µm)	3.877 ± 0.312	3.128 ± 0.4288	2.486 ± 0.2376	2.429 ± 0.2350	2.133 ± 0.1229	2.114 ± 0.1137

**Table 6:** Average COF values and their standard deviations for the different test conditions.

Sample	Not Dehydrogenated	Dehydrogenated
Ni-P	0.50 ± 0.02	0.47 ± 0.02
Ni-P/ZrO_2_	0.39 ± 0.03	0.45 ± 0.01
Ni-P/ZrO_2_/400 °C 1 h	0.42 ± 0.03	0.36 ± 0.03

## Data Availability

The data presented in this study are available upon request from the corresponding author due to institutional policy.
